# Supplementary material for: wenda_gpu: fast domain adaptation for genomic data
Source: Bioinformatics. 2022 Oct 4;38(22):5129–30. doi: 10.1093/bioinformatics/btac663 (PMC9665854; doi:10.1093/bioinformatics/btac663)
Supplement: btac663_Supplementary_Data [file btac663_supplementary_data.zip › btac663_Supplementary_Data/supplement.pdf]

Supporting Information for

wenda_gpu: fast domain adaptation for genomic data

Hippen, Crawford, Gardner, and Greene

Casey S. Greene, e-mail: casey.s.greene@cuanschutz.edu

This PDF file includes:

Supporting text
Fig. S1
Table S1
SI References

Supporting Information Text

Weighted elastic net details. The penalty in a elastic net regression is a linear combination of penalties from lasso (L1 penalty) and ridge regression (L2 penalty). This generally favors sparser solutions than linear regression, where the only penalty is the residual sum of squares (RSS). The fitted coefficients of elastic net regression can be written thus:

$$\hat{\beta} = argmin_{\beta}(RSS(\beta) + \lambda(\alpha \sum_{f=1} |\beta_f| + (1-\alpha) \sum_{f=1} \beta_f^2)) \quad [1]$$

The weighted elastic net adds an additional parameter: a matrix of feature-specific weights based on confidence scores obtained from the feature models. With this addition, the coefficients of weighted elastic net regression are optimized as:

$$\hat{\beta} = argmin_{\beta}(RSS(\beta) + \lambda(\alpha \sum_{f=1} (1-c_f)^k |\beta_f| + (1-\alpha) \sum_{f=1} (1-c_f)^k \beta_f^2)) \quad [2]$$

Here, c_f is the mean feature model confidence score across all samples in the target data. As in (1), in wenda_gpu the weight between lasso and ridge regression is treated as a design decision and set at α=0.8. An optimal value of λ is determined via cross-validation. The parameter k determines how strictly to penalize features with low confidence scores, and as in (1) we run elastic net regression for a range of k from 1 to 35. For the methylation age and TCGA mutation status tasks, we select the best k *post hoc* based on accuracy compared to true target labels.

GPyTorch implementation details. We implement our Gaussian process model using GPyTorch (2), which uses linear conjugate gradients and stochastic Lanczos quadrature to scale to the large amount of training data (3). Matrix vector multiplies with kernel matrices are implemented using the KeOps library (4), which supports training in O(n) space complexity via map-reduce computation. To minimize the number of training iterations used, we use the L-BFGS optimizer (5). Additionally, we use the batch-mode Gaussian process support of GPyTorch, which enables us to train the Gaussian processes for each feature model in parallel. Achieving this parallelism relies on the fact that GPyTorch models only access data through kernel matrix-vector multiplies, and both CUBLAS and KeOps natively supports batch-mode matrix multiplication. This enables more efficient GPU utilization in the setting where one of the Gaussian processes trained for a feature model does not fully utilize GPU resources.

While wenda_gpu specifies a linear kernel by default, other kernels can be specified. Using other kernels, such as GPyTorch's spectral delta kernel, may provide improved runtime at the expense of model expressiveness or vice versa.

Methylation age dataset. The source and target datasets were prepared using publicly available data from The Cancer Genome Atlas (TCGA) and the Gene Expression Omnibus (GEO). The source dataset contains data from 19 different tissues, most of which are blood and are aggregated for our paper. The target dataset has data from most of these tissue types, with the notable exclusion of cerebellum tissue. Originally these datasets had data for over 400,000 methylation sites, but were filtered to 12,980 methylation sites using a regular elastic net model. Chronological ages ranged from 0-103 years. These data are available at https://github.com/greenelab/wenda_gpu_paper. More details about the collection and processing of the datasets are in (1).

Runtime calculation. All models for the simulated datasets (Fig. S1A) were run using a NVIDIA Titan Xp (GP102) for the wenda_gpu runs, and using 6 Intel Core i5-8500 CPUs with 3.0 GHz for the wenda_orig and wenda_cputest runs. The speed of all packages described will vary based on the hardware used.

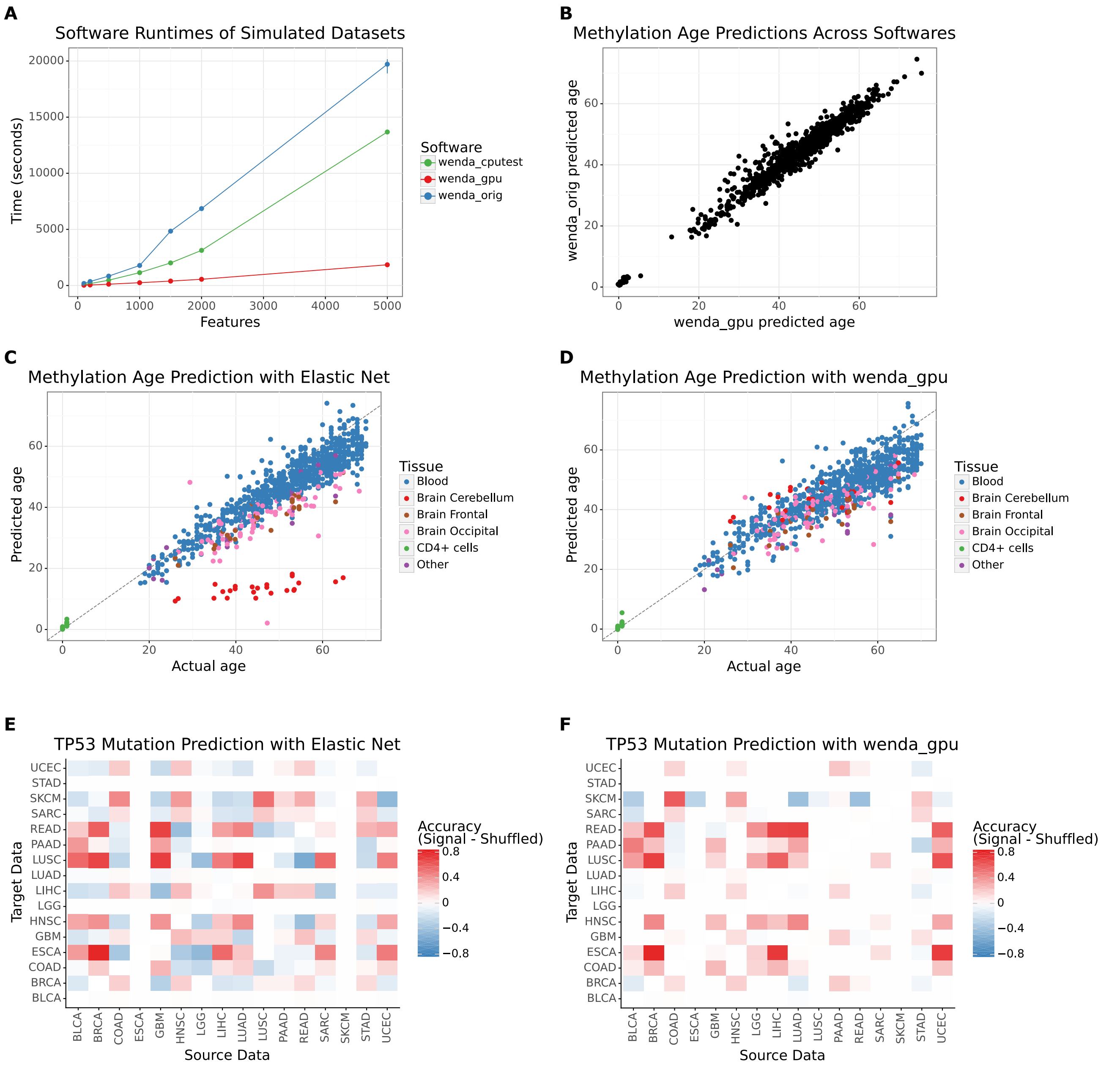

Fig. S1. (A) Mean runtimes in wenda_orig and wenda_gpu to train feature models and generate confidence scores for simulated datasets (3 replicates for each number of features). (B) Correlation between wenda_gpu and wenda_orig on methylation age prediction at k=6. X axis is one of 10 cross-validation folds from wenda_orig, chosen for being closest to median correlation value of 0.977. (C, D) Age predictions on methylation data done by (C) regular elastic net and (D) wenda_gpu, compared with actual age. With a regular elastic net, prediction is poor on cerebellum tissue (red), which is not in the source dataset. Using wenda_gpu (k=6), cerebellum samples are predicted well, even though the model was not provided with labels for this tissue type. (E, F) Difference in accuracy of TP53 mutation prediction on pairs of TCGA cancer types compared to a model with shuffled labels, done using elastic net (E) and wenda_gpu (F).

Number of features	wenda_orig	95% CI	wenda_gpu	95% CI	wenda_cputest	95% CI
100	180.33	(163.79, 196.87)	23.67	(22.23, 25.10)	93.67	(60.59, 126.75)
200	355.00	(339.49, 370.51)	45.33	(43.90, 46.77)	168.33	(163.16, 173.50)
500	835.67	(815.74, 855.59)	116.33	(114.9, 117.77)	471.33	(463.74, 478.92)
1000	1783.00	(1748.85, 1817.15)	247.67	(246.23, 249.10)	1145.00	(1131.86, 1158.14)
1500	4841.67	(4753.55, 4929.79)	393.00	(388.70, 397.30)	2012.00	(2009.52, 2014.48)
2000	6851.33	(6388.85, 7313.81)	554.33	(552.90, 555.77)	3130.00	(3112.61, 3147.39)
5000	19717.33	(17952.29, 21482.38)	1844.67	(1812.63, 1876.7)	13678.33	(13563.92, 13792.75)

Table S1. Runtimes (mean and 95% confidence interval) across simulated datasets

References

1. Lisa Handl, Adrin Jalali, Michael Scherer, Ralf Eggeling, and Nico Pfeifer. Weighted elastic net for unsupervised domain adaptation with application to age prediction from DNA methylation data. *Bioinformatics*, 35(14):i154–i163, July 2019. ISSN 1367-4803. . URL https://academic.oup.com/bioinformatics/article/35/14/i154/5529259.
2. Jacob Gardner, Geoff Pleiss, Kilian Q Weinberger, David Bindel, and Andrew G Wilson. Gpytorch: Blackbox matrix-matrix gaussian process inference with gpu acceleration. *Advances in neural information processing systems*, 31, 2018.
3. Ke Wang, Geoff Pleiss, Jacob Gardner, Stephen Tyree, Kilian Q Weinberger, and Andrew Gordon Wilson. Exact gaussian processes on a million data points. *Advances in Neural Information Processing Systems*, 32, 2019.
4. Benjamin Charlier, Jean Feydy, Joan Alexis Glaunès, François-David Collin, and Ghislain Durif. Kernel operations on the gpu, with autodiff, without memory overflows. *Journal of Machine Learning Research*, 22(74):1–6, 2021.
5. Dong C Liu and Jorge Nocedal. On the limited memory bfgs method for large scale optimization. *Mathematical programming*, 45(1):503–528, 1989.
